# Global Responses of Resistant and Susceptible Sorghum (*Sorghum bicolor*) to Sugarcane Aphid (*Melanaphis sacchari*)

**DOI:** 10.3389/fpls.2019.00145

**Published:** 2019-02-22

**Authors:** Hannah M. Tetreault, Sajjan Grover, Erin D. Scully, Tammy Gries, Nathan A. Palmer, Gautam Sarath, Joe Louis, Scott E. Sattler

**Affiliations:** ^1^Wheat, Sorghum and Forage Research Unit, USDA-ARS, Lincoln, NE, United States; ^2^Department of Agronomy and Horticulture, University of Nebraska–Lincoln, Lincoln, NE, United States; ^3^Department of Entomology, University of Nebraska–Lincoln, Lincoln, NE, United States

**Keywords:** sugarcane aphid, sorghum, plant–insect interactions, *Melanaphis sacchari*, *Sorghum bicolor*

## Abstract

The sugarcane aphid (*Melanaphis sacchari*) has emerged as a significant pest for sorghum. The use of sugarcane aphid-resistant sorghum germplasm with integrated pest management strategies appears to be an excellent solution to this problem. In this study, a resistant line (RTx2783) and a susceptible line (A/BCK60) were used to characterize the differences in plant responses to the sugarcane aphid through a series of experiments, which examined global sorghum gene expression, aphid feeding behavior and inheritance of aphid resistance. The global transcriptomic responses to sugarcane aphids in resistant and susceptible plants were identified using RNA-seq and compared to the expression profiles of uninfested plants at 5, 10, and 15 days post-infestation. The expression of genes from several functional categories were altered in aphid-infested susceptible plants, which included genes related to cell wall modification, photosynthesis and phytohormone biosynthesis. In the resistant line, only 31 genes were differentially expressed in the infested plants relative to uninfested plants over the same timecourse. However, network analysis of these transcriptomes identified a co-expression module where the expression of multiple sugar and starch associated genes were repressed in infested resistant plants at 5 and 10 days. Several nucleotide-binding-site, leucine-rich repeat (NBS-LRR) and disease resistance genes similar to aphid resistance genes identified in other plants are identified in the current study which may be involved in sugarcane aphid resistance. The electrical penetration graph (EPG) results indicated that sugarcane aphid spent approximately twice as long in non-probing phase, and approximately a quarter of time in phloem ingestion phase on the resistant and F_1_ plants compared to susceptible plant. Additionally, network analysis identified a phloem protein 2 gene expressed in both susceptible and resistant plants early (day 5) of infestation, which may contribute to defense against aphid feeding within sieve elements. The resistant line RTx2783 displayed both antixenosis and antibiosis modes of resistance based on EPG and choice bioassays between susceptible, resistant and F_1_ plants. Aphid resistance from RTx2783 segregated as a single dominant locus in the F_2_ generation, which will enable breeders to rapidly develop sugarcane aphid-resistant hybrids using RTx2783 as the male parent.

## Introduction

The sugarcane aphid (*Melanaphis sacchari*; Homoptera: Aphididae) has recently emerged as a major insect pest of sorghum (*Sorghum bicolor*) in the southern plains and southeastern United States (Armstrong et al., 2015). Sugarcane aphid, which reproduces parthenogenetically, has a worldwide distribution that includes over 30 countries and it typically occurs in regions where sugarcane (*Saccharum officinarum*) and sorghum (*Sorghum bicolor*) are intensively cultivated (Singh et al., 2004). In North America, sugarcane aphid has had a long-established association with sugarcane and was infrequently reported on sorghum (Wilbrink, 1922; Denmark, 1988; White et al., 2001). However, in 2013, this insect became a major pest (Zapata et al., 2016) of grain sorghum in Texas, Louisiana, Oklahoma, and Mississippi, which resulted in significant yield losses (Villanueva et al., 2014). Based on genotypic analyses using microsatellite markers, one predominant biotype, MLL-F, appears to be associated with the widespread outbreak on sorghum in the United States (Harris-Shultz et al., 2017; Nibouche et al., 2018). Sorghum injury from sugarcane aphid results from a reduction in plant growth, leaf chlorosis and a reduction in plant nutrients (Singh et al., 2004). At high infestation levels, honeydew excreted by the sugarcane aphid can impair plant respiration and stimulate mold growth, which also can reduce photosynthesis. In addition, the aphid-produced honeydew covers the plants with a sticky layer that impedes the harvest of both sorghum grain and forage (Bowling et al., 2016). Thus, this insect represents a newly emerging threat to sorghum production.

Aphids constitute a large superfamily of piercing and sucking insects that feed on the phloem of vascular plants and are potential pests of virtually all crops (Pollard, 2009). Aphids significantly constrain plant growth and reproduction by depleting photoassimilates and vectoring plant viruses in some systems. Aphids have a wide range of hosts, however, some species are specialists that are largely restricted to a single plant species or genus, while others are polyphagous and have much broader host ranges consisting of multiple plant families (Peccoud et al., 2010). Aphids are adapted to feeding from a single plant cell type, the phloem sieve element. During feeding, salivary stylets from their piercing-sucking mouthparts penetrate layers of cells until they contact the phloem sieve elements and begin to feed passively on sap (Tjallingii, 2006). This specific mode of feeding presents an opportunity to elucidate plant defenses against aphid herbivory.

Plants have evolved a range of mechanisms to defend against, tolerate or avoid insect herbivory. Three main mechanisms responsible for imparting resistance to insects include antibiosis, antixenosis and tolerance. Antibiosis negatively impacts a pest’s physiology through reduced growth, longevity, fecundity and survival (Painter, 1951; Smith, 2005). Antixenosis, or non-preference, is based on behavioral avoidance of a host due to a trait or set of traits that deter insects from feeding (Painter, 1951). Plant tolerance enables plants to maintain growth and productivity and avoid negative fitness impacts associated with feeding or defense responses despite harboring pest numbers similar to those observed on susceptible plants (Panda and Khush, 1995). Because tolerance does not negatively impact the physiology or the behavior of the aphid, the selective pressure for the emergence of new aphid biotypes is presumed to be limited (Koch et al., 2016). Identifying and characterizing the mechanisms of insect resistance are critical to improving our understanding of plant-insect interactions and developing novel strategies to defend plants from insect herbivory.

The use of insecticides has been a major component of integrated strategies for managing sugarcane aphid, while resistant hybrids are being developed and are also key components of management regimes. The molecular mechanisms that confer resistance to sugarcane aphid remain unknown, despite the fact that more resistant parental lines and hybrids are being developed. To characterize differences in the responses to sugarcane aphid feeding between a susceptible (A/BCK60) and resistant (RTx2783) sorghum lines, a series of experiments were performed to examine global sorghum gene expression, aphid feeding behavior and inheritance of aphid resistance. The resistant parental line used in the current study, RTx2783, was derived from ‘Capbam’ (introduced from Russia) and SC110 (from the USDA/Texas A&M sorghum conversion program), which was originally developed for resistance to greenbug biotypes C and E (Peterson et al., 1984), and recently identified as resistant to the sugarcane aphid (Armstrong et al., 2015). Understanding the differences of how susceptible and resistant plants respond to sugarcane aphid and how aphids respond to these plants will provide critical information on ways to manage this emerging threat to sorghum production.

## Materials and Methods

### Plants

Two sorghum (*Sorghum bicolor*) lines used in this study were the sugarcane aphid resistant RTx2783 line and the aphid-susceptible A/BCK60 line pair, hereafter, referred to as resistant and susceptible respectively. RTx2783 is a grain sorghum developed by the Texas Agricultural Experiment Station to have resistance to *Schizaphis graminum* (greenbug) (Peterson et al., 1984). A/BCK60 is a grain sorghum that is greenbug susceptible (Hackerott and Harvey, 1971). Crosses were made between the susceptible and resistant lines at the University of Nebraska-Lincoln greenhouse facility under a 16:8 h light:dark cycle at 29–30°C and 26–27°C during, day and night, respectively. Seeds produced from crosses were harvested and planted for F_2_ seed production. For all screening experiments described, plants were grown in Metro-mix 360 (Hummert International) in a plant growth chamber (Conviron F7, Controlled Environments Ltd) at 26°C under a 12:12 h light:dark cycle. All experiments were initiated when sorghum seedlings were 2 weeks old (the 3–4 leaf stage).

### Sugarcane Aphid Colony

The sugarcane aphid (*Melanaphis sacchari*) source colony was founded from a single aptera collected from infested *Sorghum bicolor* (L.) Moench ssp. sorghum, at the Louisiana State Agricultural Center Dean Lee Research Station, Alexandria, LA, in July 2014, designated LSU-SCA14, and was the source for aphids used in the following experiments. A single parthenogenic female of *M. sacchari* from the above colony was reared on the susceptible sorghum genotype. The progeny from this single female were maintained by transfer to a susceptible sorghum genotype, BCK60 every 2 weeks in a plant growth chamber (Conviron F7, Controlled Environments Ltd.) under a 12:12 h light:dark cycle; temperatures were maintained at 26°C. As needed apterous aphids were transferred to experimental plants with a fine-bristled paintbrush.

### Open Tray Resistance and Susceptibility Screens

Seeds for each sorghum line were planted in a randomized block design in two replicated 96-cell trays, with a total of 10 susceptible, 10 resistant, 10 F_1_ and 162 F_2_ plants. Each plant was infested with 3–5 sugarcane aphids and subsequently monitored for aphid damage by two independent observers for up to 19 days post infestation. Plant damage was rated by amount of leaf discoloration and leaf rolling. Specifically, the amount of damage was quantified using a 1–5 scale adapted from Heng-Moss et al. (2002) (1-plants appear healthy, may have small spots of discoloration; 2-discoloration and leaf rolling comprising 20–39% of total leaf area; 3-discoloration and leaf rolling that comprised 40–59% of total leaf area; 4-discoloration and leaf rolling that is 60–79% of total leaf area; 5-plants appear dead, discoloration and leaf rolling that is 80–100% of total leaf area; Supplementary Figure S1). Ratings from the two independent observers were averaged to obtain a single damage score per day. Damage scores from days 7 through 19 were used to calculate the rate of damage change, which was represented by a linear slope for each individual plant (Supplementary Figure S2). A χ^2^ test was performed to determine whether observed results for F_2_ plants fit a 3 (resistant) to 1 (susceptible) segregation ratio, indicative of a dominant mode of inheritance.

### Caged Plants for Evaluation of Sugarcane Aphid Damage (No-Choice Assay)

Seedlings were screened in a no-choice assay to determine aphid survival and plant damage for a period of up to 15 days post infestation. Seeds were planted in cone-tainers (Ray Leach SC10; Hummert International) and randomized in a cone-tainer rack. Individual plants were infested with 3 aphids per plant and caged with tubular plastic cages with vents covered with organdy fabric to confine sugarcane aphids. Four cone-tainers of each genotype (resistant, susceptible and F_1_) were not infested and served as controls to ensure aphids did not move among cages. The numbers of aphids per plant were counted and plant damage scored at 5, 8, 10, 12, 13, 14, and 15 days post infestation (*n* = 4 replicates per timepoint). Plant damage was rated as described earlier (Supplementary Figure S1).

### Sugarcane Aphid Preference (Choice Assay)

To examine sugarcane aphid preference, resistant, susceptible and F_1_ plants were grown in pots (9 cm in diameter by 9 cm in depth). One seed from each genotype was planted near the perimeter of the pot and arranged in such a manner that it was equally spaced from other seeds and the center of each pot (approximately 7 cm between plants and 3.5 cm from the center) with six replicates. Fifty apterous adults were transferred to a plastic weigh boat in the center of each pot, which was placed approximately 1.0 cm away from each plant. Pots were arranged within a plastic flat filled with water to prevent aphids from moving between pots. The numbers of adult and nymph aphids were counted on each sorghum genotype at 24 and 48 h post aphid introduction.

### EPG Recording

For the feeding behavior study, plants were grown in cone-tainers (Ray Leach SC10; Hummert International) filled with Metro-mix 360 and were maintained as previously described for the aphid and plant performance experiment. Sorghum plants with uniform growth were selected for EPG experiments (Reese et al., 2000; Tjallingii, 2006; Louis et al., 2012), conducted in a laboratory conditions at 22–24°C and 40–45% relative humidity (RH) under continuous light conditions. Adult aphids were starved for 1 h, prior to EPG recording. A gold wire attached to the brass nail (insect electrode) was glued to dorsum of aphids using a silver conductive glue. The plant electrode (a stiff copper wire) was inserted into potted soil. A GIGA-8 EPG system^1^ (W.F. Tjallingii, Wageningen, Netherlands) with a 10^9^ Ω resistance amplifier was connected to both plant and insect electrodes and an adjustable plant voltage was used for assessing feeding behavior of sugarcane aphids on different sorghum genotypes (resistance, susceptible and F_1_). The insect electrodes were inserted into the EPG probe and the wired aphids were carefully placed on the sorghum leaf. Recordings were made for eight plants (mix of three selected genotypes) at a time, which were placed randomly in a Faraday’s cage. EPG recordings were obtained from 16 replications per genotype for 10 h. EPG acquisition software, *Stylet^+^* (EPG Systems, Wageningen, Netherlands) was used to record waveforms for aphids feeding on sorghum genotypes.

EPG waveforms were categorized into four phases: Pathway phase, xylem phase, sieve element or phloem phase and non-probing phase. The pathway phase represents penetration and withdrawal of stylets intercellularly. Number of potential drops per 10 h waveform recording was also calculated, which reflects brief intracellular punctures. The xylem and phloem phases represent ingestion of water and phloem sap, respectively. The non-probing phase shows the period of relatively no stylet movement. The other parameters calculated from the EPG waveforms include time to first probe by aphid during the 10 h recording (time difference between starting of recording and first insertion of stylet into plant) and the time to first sieve element phase.

### Plant Transcriptional Response to *M. sacchari* Feeding

Seeds were grown in cone-tainers and randomized within cone-tainer racks as described above. The plants were arranged in a 2 × 2 × 3 factorial design consisting of two treatments (infested and control), two genotypes (resistant and susceptible), three harvest timepoints (5, 10, and 15 days post infestation) and three replicates for all treatment and time combinations. Five sugarcane aphids were initially placed on ‘infested’ plants at day 0. Infested and control plants were individually caged in the same manner described for the damage studies. At each harvest date, aphid numbers was first counted, aphids were then brushed off, and all leaves present on the plant were collected, immediately flash frozen in liquid nitrogen and stored at -80°C for future processing.

Total RNA was extracted from three biological replicates per time point and treatment. A total of 36 RNA samples were extracted (3 harvest dates × 3 replications × 2 treatments × 2 genotypes). RNA was extracted according to Suzuki et al. (2004). RNA was treated with DNase on-column (Zymo Research). RNA quality was assessed using an Agilent 2100 Bioanalyzer (Agilent Technologies) and two micrograms of total RNA per sample were utilized for TruSeq^TM^ library preparation and RNA sequencing on an Illumina HiSeq2500 platform, generating 100 bp single-end reads. The barcoded libraries were multiplexed and sequenced across two lanes at the University of Nebraska Medical Center DNA Sequencing Core Facility, Omaha, NE, United States ^2^.

High quality Illumina reads were mapped to the *S. bicolor* genome v3.1^3^ using HISAT2 v2.0.5 (Kim et al., 2015) with default parameters. Files containing mapped reads were sorted and formatted for downstream analysis using SAMtools v1.3.1 (Li et al., 2009) and the Subread v1.5.1 program *featureCounts* (Liao et al., 2014) was used to generate a count matrix. Differential expression analyses were performed using DESeq2 package v1.14.1 (Love et al., 2014) implemented in R v3.3.2 (R Development Core Team, 2018). A principal components analysis was also used to depict the relationships among the timepoints and replicates. Count data for statistical analysis were normalized using DESeq2 default settings and a variance stabilizing transformation was applied to correct for heteroscedasticity (Love et al., 2014). Genes expressed at low-levels were removed from the count matrix and differentially expressed genes (DEGs) for infested relative to control within each genotype and day were identified at a FDR adjusted *p*-value ≤ 0.05 and a fold change ≥ 2.0 using Wald tests (Love et al., 2014). Gene annotations from *S. bicolor* v3.1 genome were retrieved from Phytozome^3^ and matched to the expressed genes using R scripts. Weighted gene co-expression network analysis (WGCNA, version 1.43) (Langfelder and Horvath, 2008) was used to identify groups of DEGS with similar expression patterns across the genotypes, timepoints and treatment (Scully et al., 2018). Briefly, the following parameters were used with the blockwiseModules function: TOMtype = “signed,” mergeCutHeight = 0.25, minModuleSize = 30. Pathway analysis was conducted utilizing the KEGG pathway database^4^; KEGG Orthology (KO) numbers obtained from Phytozome gene annotations were used for mapping to the ‘sbi’ (*S. bicolor)* KEGG reference metabolic pathway. Raw counts of genes that were differentially expressed (FDR ≤ 0.05 and log-fold change ≥ 1.0) were log-transformed and *Z*-score standardized. Heatmaps were prepared using hierarchical clustering using the ‘Ward’ method implemented in JMP 12.2.0 (SAS Institute Inc.). Green represents low expression level and magenta represents high expression level. C = control plants; I = infested plants; S = susceptible (BCK60); R = resistant (RTx2783). The raw Illumina reads for this study can be found at NCBI Sequence Read Archive database with accession number PRJNA492261.

### Statistical Analysis

Statistical analysis of the results from the caged plants for evaluation of sugarcane aphid damage, sugarcane aphid preference (choice assay) and EPG recordings evaluations were performed using analysis of variance in JMP v12.2.0 (SAS Institute Inc.). Data were tested for normality using the Normality function in JMP and were log transformed if the data failed to meet normality. For different EPG parameters, the data were analyzed using one-way analysis of variance (ANOVA), implemented in PROC GLIMMIX in SAS 9.4 (SAS Institute, Cary, NC, United States). *Post hoc* pairwise comparisons were performed using Tukey’s Honest Significant Differences test at α ≤ 0.05. Values presented are least square means and standard error.

## Results

### Transcriptional Response of Susceptible Sorghum Lines to Sugarcane Aphid

**FIGURE 1:**
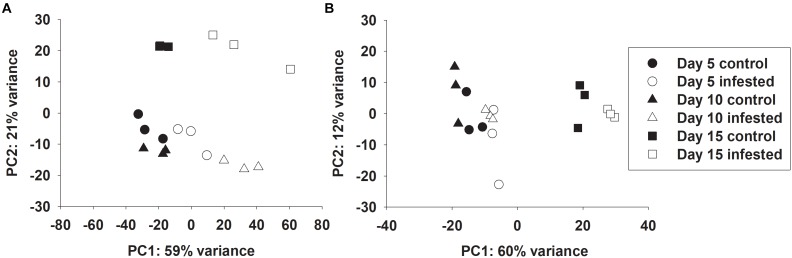
Principal component analysis of RNA-seq data on individual samples from control and infested **(A)** susceptible (BCK60) and **(B)** resistant (RTx2783) sorghum lines at 5, 10, and 15 days post infestation.

**FIGURE 2:**
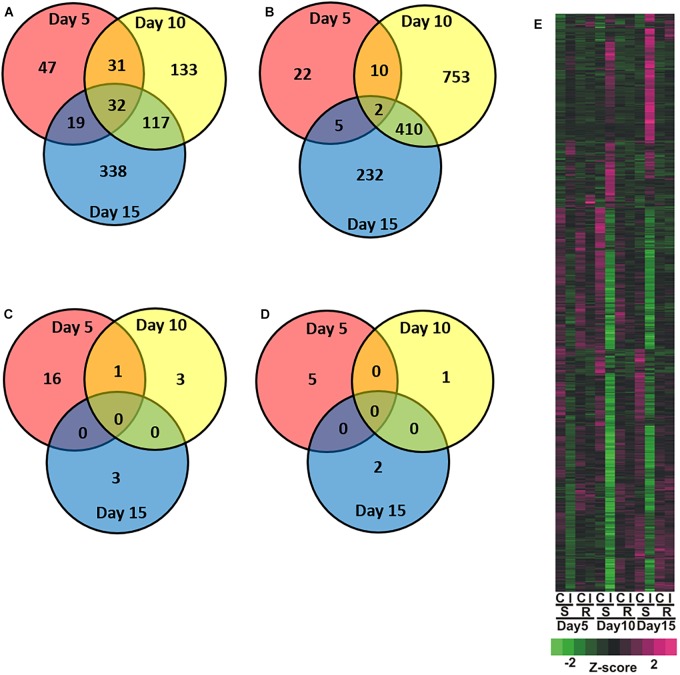
Overview of global changes in differentially expressed genes across days for the sugarcane infested resistant (RTx2783) or susceptible (BCK60) sorghum line relative to control non-infested resistant (RTx2783) or susceptible (BCK60) sorghum line. Venn diagrams of susceptible **(A)** increased and **(B)** decreased and resistant **(C)** increased and **(D)** decreased genes detected in infested relative to control. **(E)** Heatmap analysis of differentially expressed genes in control (C), infested (I), for susceptible (BCK60; S), resistant (RTx2783; R) within timepoint. Numbers within regions in Venn diagram indicate common and unique genes within each sector.

Global transcriptional responses of resistant and susceptible sorghum plants to sugarcane aphid infestation over the course of 15 days were identified using RNA-seq. Principal component analysis (PCA) effectively separated the different transcriptome samples by treatment and time for the susceptible line (Figure 1A), which indicated the changes in transcriptome profiles over the 15 days time course arose from both development and stress associated with aphid-feeding. The first principal component (PC1, which accounted for 59% of the variance; Figure 1A) differentiated the transcriptomes of the infested and uninfested susceptible plants. The transcriptomes of the control and infested susceptible plants 15 days post infestation were differentiated from the transcriptomes of the 5 and 10 days counterparts by the second principal component (PC2; accounted for 21% of the variance), which likely accounted for developmental changes (Figure 1A). DEGs were identified for sugarcane aphid infested plants relative to uninfested plants within timepoint using an FDR ≤ 0.05 and a log fold change (LFC) of ≥ 1.0. For susceptible plants, the maximum number of DEGs occurred at 10 days post infestation (1,488) relative to uninfested controls within timepoint. Smaller numbers of DEGs were observed at 5 (168) and 15 (1,155) days post infestation for the infested relative to uninfested plants (Figure 2A,B,E and Supplementary Tables S1, S2).

**FIGURE 3:**
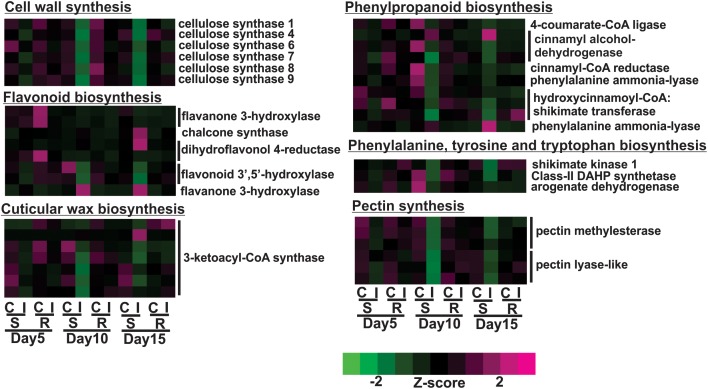
Differentially expressed genes associated with cell walls in susceptible (BCK60) and resistant (RTx2783) sorghum lines for sugarcane aphid infested plants relative to controls for days 5, 10, and 15.

The susceptible line, BCK60, had a “typical” response to herbivory marked by early vigorous defensive responses, but the plant was unable to sustain these responses and ultimately died. Over the time course, the susceptible line exhibited decreased gene expression for cell wall associated genes with sugarcane aphid infestation, which was most pronounced at 10 days post infestation. For example, cellulose synthase A (CesA) genes, which encode the protein complex responsible for the synthesis of the cell-wall polysaccharide cellulose, were differentially expressed in the susceptible infested plants relative to uninfested controls. The expression of six of the eleven sorghum CesA genes were down-regulated on days 10 and 15 in infested susceptible plants (Figure 3). At the same time, the expression of genes involved in the phenylpropanoid biosynthesis pathway were also down-regulated at days 10 and 15, which included genes encoding for 4-coumarate-CoA ligase (4CL), cinnamyl-CoA-reductase (CCR), cinnamyl alcohol dehydrogenase (CAD), phenylalanine ammonia lyase (PAL) and hydroxycinnamoyl CoA:shikimate transferase (HCT) (Figure 3). In contrast, the expression of the genes encoding one PAL and one CAD were induced at day 10 and remained up-regulated at day 15 in the infested susceptible line relative to the uninfested controls (Figure 3). The expression of several flavonoid biosynthetic genes were also induced in the susceptible line at 15 days post infestation relative to uninfested plants; these genes encoded a chalcone synthase (CHS), a dihydroflavonol 4-reductase (DFR) and a flavanone 3-hydroxylase (F3′H) (Figure 3). The expression of two sorghum genes similar to flavonoid 3′-5′hydroxylase (F3′5′H) were decreased in infested susceptible plants relative to uninfested control plants at day 10 (Figure 3). The expression levels of genes that encode shikimate pathway enzymes, which synthesize the aromatic amino acid substrates of phenylpropanoid pathway, were decreased in the susceptible line at days 10 and 15 post infestation. These genes encoded the following enzymes: phospho-2-dehydro-3-deoxyheptonate aldolase (DAHP), shikimate kinase-1 (SK1) and arogenate dehydrogenase 1 (ADT; Figure 3). In the sugarcane aphid-infested susceptible plants relative to uninfested controls, the expression of genes involved in cuticular wax biosynthesis were also altered, which included six 3-ketoacyl-CoA synthase (KCS) genes that were down-regulated at day 10 and one KCS that was up-regulated at day 15 (Figure 3). Pectin methylesterase (PME) and pectin lyases (PEL) genes, showed decreased expression starting at day 5 with the lowest expression levels at day 10 post aphid infestation (Figure 3). Sugarcane aphid infestation appears to trigger decrease cell wall synthesis and direct phenylpropanoid metabolism away from lignin toward flavonoids synthesis by 10 days post infestation in susceptible plants.

**FIGURE 4:**
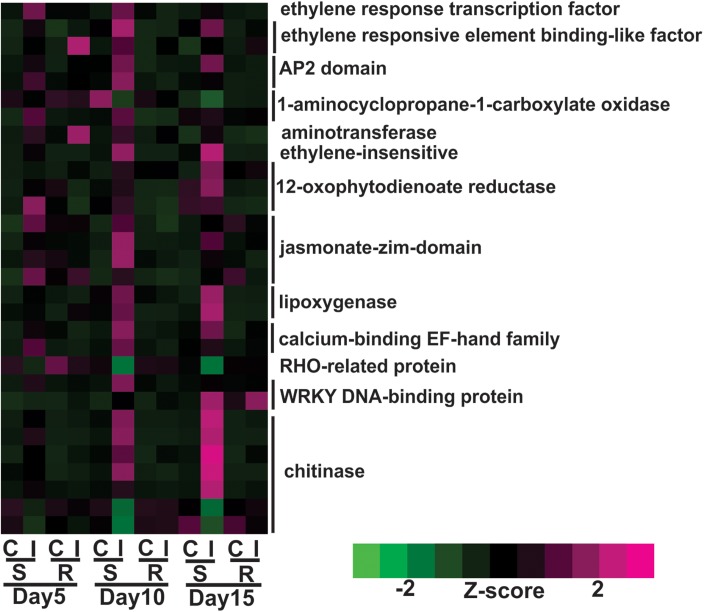
Differentially expressed genes associated with plant defenses and phytohormone metabolic pathways for sugarcane aphid infested susceptible (BCK60) and resistant (RTx2783) sorghum lines relative to the respective controls at day 5, 10, and 15.

Expression of genes encoding jasmonic acid (JA) and ethylene phytohormones was increased in the susceptible plants following aphid infestation. Expression levels of nine genes associated with JA metabolism were significantly increased during sugarcane aphid infestation in the susceptible sorghum line at day 5, and their expression levels were more pronounced at day 10 post infestation, but significant differences in gene expression between control and infested plants of the resistant line were not observed (Figure 4). These genes encoded enzymes 12-oxophytodienoate reductase, lipoxygenase-9 and jasmonate-zim-domain genes (Figure 4). Genes associated with ethylene metabolism were also differentially expressed in the susceptible line upon aphid infestation. In the susceptible line, the expression of genes encoding an ethylene response transcription factor and a 1-aminocyclopropane-1-carboxylate oxidase were induced at day 5 and was an early transcriptional response to aphid infestation (Figure 4). By day 10, the expression of other ethylene synthesis related genes were also induced in aphid infested susceptible lines. The expression of two genes encoding WRKY transcription factors were found to be up-regulated after aphid infestation, one of which was increased in the susceptible genotype at day 10 and the other was increased at day 15 (Figure 4). The expression of chitinase-related genes was also increased by day 10 and at day 15 in the aphid infested susceptible plants relative to uninfested control plants (Figure 4).

**FIGURE 5:**
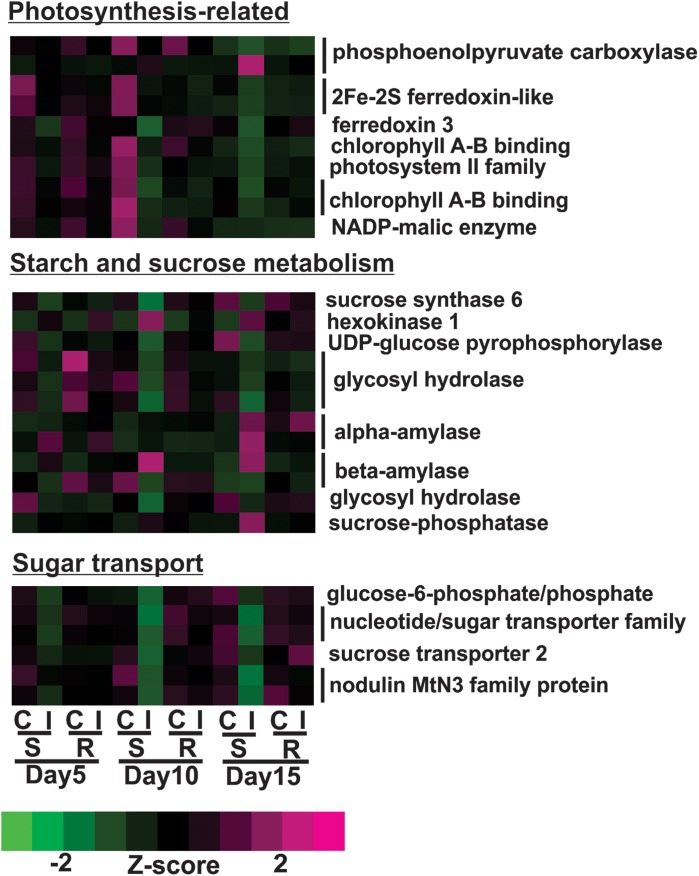
Differentially expressed genes associated with photosynthesis, starch and sugar metabolism and sugar transport for sugarcane aphid infested susceptible (BCK60) and resistant (RTx2783) sorghum lines relative to controls for day 5, 10, and 15.

Transcriptional profiles of genes associated with photosynthesis shown in Figure 5 were generally down-regulated in infested plants relative to uninfested plants at day 5 in susceptible plants. By day 10, the expression levels of these genes in infested plants decreased further in susceptible plants compared to the previous time point. This group included genes encoding for three chlorophyll A-B binding proteins, two ferredoxin-like proteins, two photosystem II proteins and two phosphoenolpyruvate carboxylases (Figure 5). Sugarcane aphid herbivory also affected the expression of genes related to sugar and carbohydrate metabolism in the susceptible line (Figure 5). Specifically, the expression of genes, including sucrose synthase and UDP-glucose pyrophosphorylase genes assigned to the starch and sucrose metabolism KEGG pathway (K01087), were down-regulated in the aphid infested susceptible line relative to uninfested susceptible at day 10. In contrast, the expression levels of other starch and sucrose metabolism genes, including genes encoding for alpha-amylase (two copies), beta-amylase, hexokinase and sucrose phosphatase were increased in days 10 or 15 post infestation in the susceptible line (Figure 5). These transcriptional changes indicate potential shift in starch and sucrose metabolism to maintain sucrose in equilibrium when faced with reduced photosynthetic capacity in the infested susceptible plants. Similarly, the expression of genes related to sugar transport were down-regulated in susceptible, infested plants relative to uninfested plants as early as day 5, which may indicate that aphid-feeding induce a shut-down of sugar export due to reduced photosynthetic capacity (Figure 5).

**FIGURE 6:**
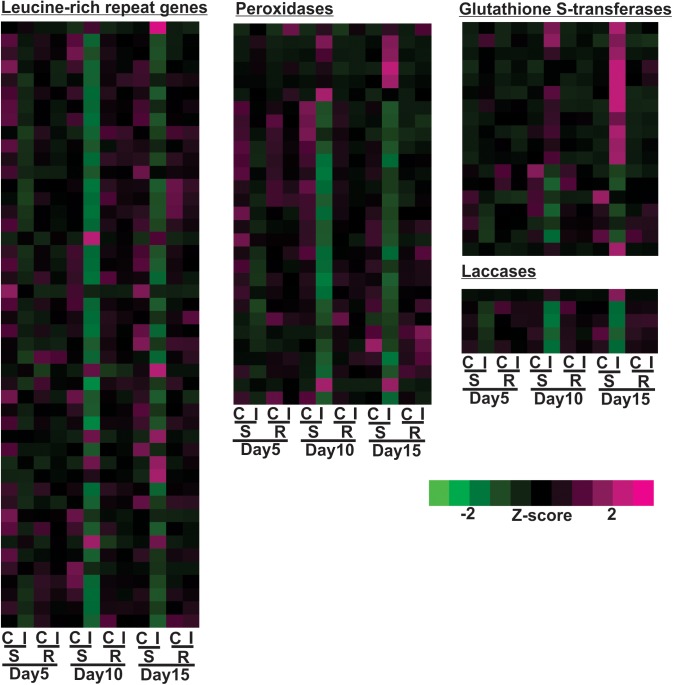
Differentially expressed genes associated with leucine-rich repeat genes, peroxidase, glutathione S-transferases (GSTs) and laccases for sugarcane aphid infested susceptible (BCK60) and resistant (RTx2783) sorghum lines relative to control plants for day 5, 10, and 15.

Expression profiles of large groups of genes associated with plant defense, such as nucleotide-binding-site leucine-rich-repeats (NBS-LRR), peroxidases, glutathione S-transferases and laccases, are shown in Figure 6. 15% of the differentially expressed NBS-LRRs were increased in the susceptible line after aphid infestation by day 10 or 15, while 85% of the differentially expressed NBS-LRRs were down regulated, a much larger portion. Similar to NBS-LRRs, 24% of the differentially expressed peroxidases were up-regulated in the susceptible line, but 76% of them were decreased at either 10 or 15 days post infestation relative to uninfested control (Figure 6). A large portion of the differentially expressed glutathione S-transferases (GSTs) (67%) were up-regulated at day 15 and some of them as early as day 10, while 33% of them were down-regulated in the infested, susceptible plants at day 10 (Figure 6). Aphid infestation in the susceptible line results in decreased gene expression for a large number of laccases in as early as 5 days, and lowest expression levels were observed at 10 days (Figure 6). The expression of only one laccase gene was up-regulated at day 15 in the aphid-infested susceptible line. Overall, aphid infestation induced global gene expression changes in the susceptible line that were indicative of high levels of herbivory-related stress and cessation of plant growth.

### Transcriptional Response of Resistant Sorghum Lines to Sugarcane Aphid

Principal component analysis separated the transcriptomes of resistant sorghum line by time point and partially by aphid treatment (Figure 1B), which suggests that development-related changes had a greater impact on the transcriptional profiles than sugarcane aphid infestation over the time course. At 15 days post infestation, resistant plants were differentiated from 5 and 10 days plants by the first principal component (PC1, which accounted for 60% of the variance), and by day 5 the transcriptomes from infested plants were partially differentiated from uninfested control plants. Principal component 2 (PC2, which accounts for 12% of the variance) partially differentiated 5 and 10 days infested from uninfested control plant transcriptomes (Figure 1B). Global changes in DEGs were identified using an FDR ≤ 0.05 and a LFC of ≥ 1.0. For resistant plants, there were 22, 5 and 5 genes differentially expressed at days 5, 10 and 15, respectively, for the infested plants relative to uninfested plants within each time point (Figure 2C–E and Supplementary Tables S1, S2).

Overall, the resistant line exhibited few changes in the gene expression levels between aphid infested and uninfested plants. Genes related to cell wall synthesis, photosynthesis, phytohormones and carbohydrate metabolism were all differentially expressed in the susceptible line upon infestation. In contrast, the expression levels of these genes were relatively unaffected in the resistant plants upon aphid infestation. The expression of flavonoid biosynthetic genes, two F3′H and one DFR were down-regulated in the aphid-infested resistant plants at day 5 relative to uninfested ones (Figure 3). Increased gene expression of an ethylene responsive element binding-like factor gene was observed at day 5 after aphid infestation exclusively in the resistant plants (Figure 4). Gene expression levels related to plant defense increased in the susceptible plants post aphid infestation relative to uninfested control plants. However, this transcriptional response was not observed in the resistant plants. The same WRKY transcription factor that was up-regulated in the susceptible line at day 15 post aphid infestation was also up-regulated in the resistant line (Figure 4). Generally, genes involved in photosynthesis were down-regulated in infested plants relative to uninfested plants at day 5 for the resistant plants (Figure 5). However, by day 10, the expression levels of photosynthesis related gene expression did not differ between the infested and uninfested control for the resistant plants.

### Network Analysis Identifies Sets of Genes Associated With Resistant and Susceptible Responses to Sugarcane Aphid

**FIGURE 7:**
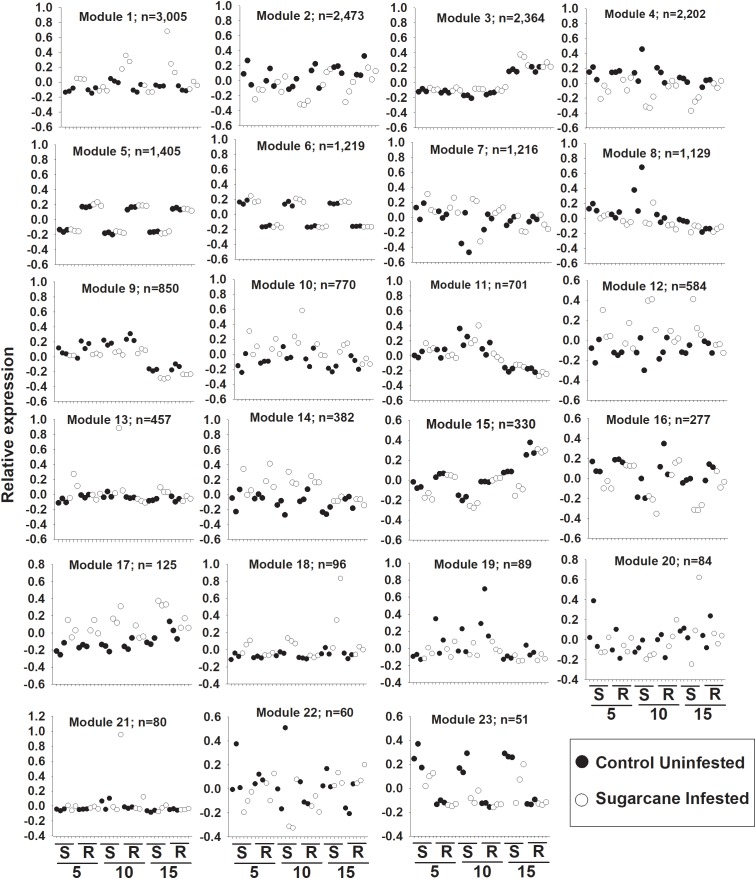
Weighted gene co-expression network analysis (WGCNA) of differentially expressed genes in susceptible (S; BCK60) and resistant (R; RTx2783) sorghum plants infested with sugarcane aphids. Expression patterns of genes assigned to co-expression module. 5 = day 5, 10 = day 10 and 15 = day 15 post infestation.

Weighted gene co-expression network analysis (WGNCA) further illustrated the impacts of sugarcane aphid on the susceptible line and resistant line (Supplementary Table S3). Four out of a total of 23 co-expression modules (Module 1, 12, 18, and 23) identified were associated specifically with the susceptible infested plants (Figure 7). While another six out of the 23 co-expression modules (Module 4, 9, 10, 14, 16, and 17) identified were associated with both the resistant and susceptible lines during sugarcane aphid infestation (Figure 7). Module 4, 9, and 16 consisted of 2202, 850, and 277 genes, respectively, whose expression was decreased in both resistant and susceptible plants with sugarcane infestation. In contrast, modules 10, 14, and 17 consisted of 770, 382, and 125 genes, respectively, whose expression were increased in both resistant and susceptible with sugarcane aphid infestation (Figure 7). Module 14 contained genes that respond similarly across day, treatment and genotype. Module 10 and 17 contained genes that increased at day 5 for both resistant and susceptible plants with aphid infestation, however, at day 10 and 15 relative expression in the susceptible line continued to increase with aphid infestation, while the resistant line decreased to the levels of its uninfested counterparts. Module 10 contained the wound-responsive gene (Sobic.006G231200) and the ethylene responsive element binding-like factor gene (Sobic.004G296900); Module 14 contained the other wound-responsive gene (Sobic.006G231332) whose expression differentially increased in the resistant line upon aphid infestation. Module 10 and 14 contained an additional ethylene responsive element binding factor gene, a wound-responsive gene, a phloem protein 2 gene (Sobic.006G184300), two pathogenesis related genes, several NBS-LRRs, multiple peroxidases and several WRKY transcription factors. The genes in Module 9 exhibit decreased expression in both resistant and susceptible aphid infested plants, however their expression was decreased in infested resistant plants at day 5, but this response was until day 10 in susceptible plants (Figure 7). The genes in Module 9 were predominantly assigned to carbon metabolism, plant hormone signal transduction, starch and sucrose metabolism, glycolysis, and carbon fixation KEGG pathways (Supplementary Table S3). The genes in Module 4 and 16 were predominantly related to phenylpropanoid biosynthesis, carbon metabolism, amino sugar metabolism and nucleotide sugar metabolism, while the genes in module 14 and 17 were related to plant hormone signal transduction and starch & sucrose metabolism KEGG pathways. Module 1, 10, 12, and 18 consisted of 3005, 770, 584, and 96 genes, respectively, whose expression was up-regulated in infested susceptible plants (Figure 7), whereas module 23 consisted of 51 genes down-regulated in infested susceptible plants (Figure 7). Module 1 genes were predominantly related to phenylpropanoid biosynthesis, plant hormone signal transduction and plant-pathogen interaction KEGG pathways. Module 10 and 12 contained genes related to plant hormone signal transduction and plant-pathogen interaction KEGG pathways and module 18 contained genes related to phenylpropanoid biosynthesis KEGG pathways. While the gene profiles in Module 23 were specific to susceptible plants, and included genes related to phenylpropanoid biosynthesis, carbohydrate metabolism and plant hormone signal transduction KEGG pathways. Although sugarcane aphid infestation minimally impacted global gene expression for the resistant line, WCGNA identified expression patterns of phenylpropanoid biosynthesis, carbon metabolism, plant hormone signal transduction, carbohydrate metabolism genes that were similar to the susceptible line.

### Transcriptional Differences Between Resistant and Susceptible Control Plants

Principal component analysis effectively separated the different transcriptome samples by resistant and susceptible (Supplementary Figure S3A), which indicated genetic differences between the two lines had a large effect on transcriptome profiles over the 15 days time course. The first principal component (PC1, which accounted for 78% of the variance; Supplementary Figure S3A) differentiated the transcriptomes of the resistant and susceptible. The transcriptomes of the day 15 plants were differentiated from the transcriptomes of the day 5 and 10 plants by the second principal component (PC2; accounted for 9% of the variance; Supplementary Figure S3A transcriptomes of the uninfested susceptible and resistant plants were compared within timepoints to determine basal differences between these lines. DEGs were identified for susceptible control plants relative to resistant control plants within timepoint using an FDR ≤ 0.05 and a LFC of ≥ 1.0. There were 1,278, 1,250, and 1,051 DEGss between day 5, 10, and 15 plants, respectively (Supplementary Figures S3B,C and Supplementary Table S4).

At all three timepoints there were differences between control plants in the expression of genes encoding WRKY transcription factors, genes associated with plant defense, such as NBS-LRRs and disease resistance proteins, and genes involved in the synthesis of JA and ethylene (Supplementary Table S4). At the day 5 timepoint, the resistant line had nine genes encoding WRKY transcription factors and four genes encoding jasmonate signaling factors whose expression was increased compared to the susceptible line. At day 10, there were 72 genes encoding NBS-LRRs with increased expression in the resistant line compared with the susceptible line. Approximately 50 and 25% of the NBS-LRRs were found on chromosome 5 and 8, respectively. Day 10 and 15 had higher expression of genes involved in the synthesis of JA in the susceptible control plants over the resistant plant (Supplementary Table S4). Overall, there were differences in the transcriptomes for the susceptible and resistant control plants, which illustrated basal differences between the two lines.

### Evaluation of Sugarcane Aphid Damage on Caged Plants

**FIGURE 8:**
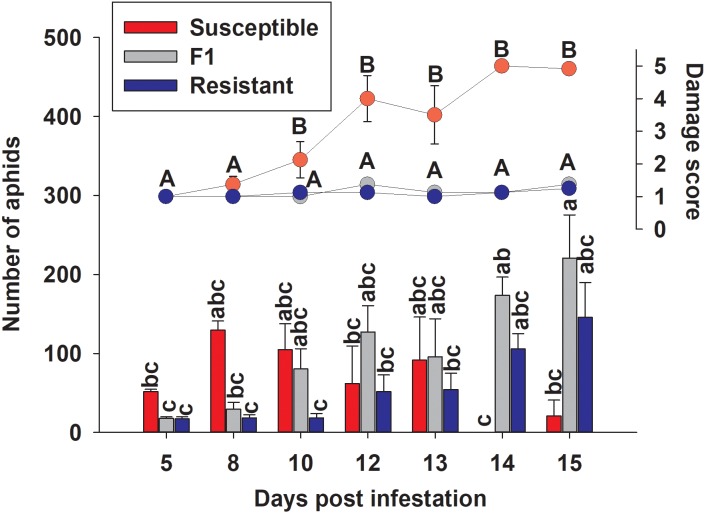
Number of sugarcane aphids and damage scores for susceptible (BCK60), resistant (RTx2783) and F_1_ (ACK60 x RTx2783) sorghum plants in a caged, no-choice assay. Values presented are least square means (±1 SE). Samples with the same letter in the same case are not significantly different from one another at α ≤ 0.05 using Tukey’s HSD test.

To examine the effect of sugarcane aphid on the plants and aphid survival, susceptible, resistant and F_1_ hybrid plants were infested with adult sugarcane aphids under cages in a no-choice assay over 15 days. Damage assessments were based on the severity of leaf symptoms that included chlorosis and leaf-rolling, while the numbers of aphids counted on each plant included both adults and nymphs (Figure 8). There was a significant genotype x day interaction for both plant damage and number of aphids (*p* < 0.0001; Table 1) and plant damage and aphid numbers differed between the lines over time (Figure 8). At 15 days post aphid infestation the resistant and F_1_ plants supported a greater number of aphids, 145.5 ± 44.2 and 220.8 ± 54.7 aphids, respectively, while the susceptible line supported only 20.8 ± 20.1 aphids (Figure 8). Furthermore, at 15 days post infestation, the resistant and F_1_ hybrid plants had significantly lower damage ratings than the susceptible plants at 1.3 ± 0.1, 1.4 ± 0.1, and 4.9 ± 0.1 respectively (Figure 8). Initially, the susceptible plants support a greater number of aphids as plant health declined aphid numbers declined. Sugarcane aphid numbers decreased on the susceptible plants after 8 days post infestation (Figure 8), because the increasing damage led to senescence/death of host likely limited aphid numbers.

**Table 1:** ANOVA results for plant damage and sugarcane aphid numbers on susceptible (BCK60), resistant (RTx2783), and F_1_ (ACK60 × RTx2783) sorghum genotypes in a no-choice assay.

	Source	*df*	Sum of Squares	*F*	*p*
Plant damage score	Genotype	2	3.04	4.14	0.0204
	Day	6	25.66	11.65	<0.0001
	Day^∗^Genotype	12	36.20	8.22	<0.0001
Number of aphids	Genotype	2	15902.00	2.35	0.1039
	Day	6	68541.45	3.37	0.0060
	Day^∗^Genotype	12	172405.62	4.24	<0.0001


*df, degrees of freedom.*

### Inheritance of Sugarcane Aphid Resistance

**FIGURE 9:**
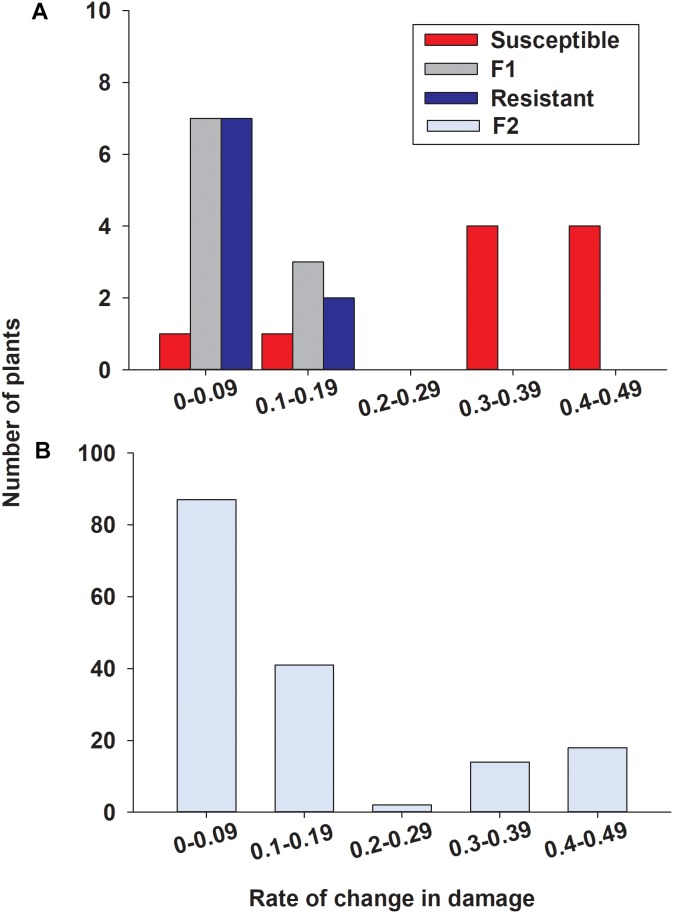
Distribution for rate of change in damage over days in **(A)** susceptible (BCK60), resistant (RTx2783), F_1_ (ACK60 x RTx2783), and **(B)** F_2_ sorghum seedlings in response to sugarcane aphid feeding. Rate of change values were determined by the slope from a linear regression fit to damage scores from 7 to 19 days post infestation for each individual (Supplementary Figure S2). Individuals in the F_2_ progeny were categorized as resistant in bins 0–0.09 and 0.1–0.19 and susceptible in bins 0.3–0.39 and 0.4–0.49.

To determine the inheritance of sugarcane aphid resistance, the susceptible (A/BCK60) and resistant (RTx2783) sorghum lines were cross-pollinated and screened for resistance in the F_2_ generation. Seedlings infested with sugarcane aphids were evaluated for damage over 19 days (Supplementary Figure S2), a rate of change in damage over days was calculated for each individual and assigned a bin category (0–0.09, 0.1–0.19, 0.2–0.29, 0.3–0.39, and 0.4–0.49; Figure 9). Resistant and F_1_ plants responded similarly with a low rate of change in damage consisting of 9 and 10 individuals, respectively, in the two lower bins ranging from 0 to 0.19 (Figure 9A) (one resistant plant died prior to aphid infestation). The susceptible sorghum line in this study had 8 individuals in the two upper bins with high rates of change in damage per day ranging from 0.3 to 0.49; however, the rates of damage of the other two BCK60 plants were similar to the resistant and F_1_ hybrid plants (Figure 9A). Overall, both RTx2783 and F_1_ plants were highly resistant based on the relatively small amount of sugarcane aphid induced damage observed over the time course. Conversely, BCK60 was shown to be highly susceptible to sugarcane aphid based upon the greater amounts of aphid induced damage observed and the greater rate of change in damage than the resistant line and F_1_ hybrids.

In the F_2_ progeny, the distribution for the rate of change in damage appeared bimodal with most of the plants skewed toward lower rates ranging from 0 to 0.19 and another smaller group of plants with rates ranging from 0.3 to 0.49, (Figure 9B). Based on the response to sugarcane aphid infestation for the susceptible and resistant parental lines, bins 0–0.19 and 0.3–0.49 were used to define F_2_ plants as resistant or susceptible, respectively (Figure 9A). In the F_2_ progeny, there were only two plants with intermediate rates of change in damage (0.2–0.29) relative to the parental lines; therefore these individuals could not be classified as resistant or susceptible and were excluded from the chi-square analysis. The segregation pattern in the F_2_ progeny for rate of change in damage from sugarcane aphid fit a 3 (128 plants resistant): 1 (32 plants susceptible) ratio (χ^2^ = 2.13, *p =* 0.144), which indicated that a single dominant locus is responsible for sugarcane aphid resistance in RTx2783 (Table 2 and Figure 9B).

**Table 2:** Observed and expected rate of change in damage scores over 19 days post aphid infestation for F_2_ [ACK60 (susceptible) × RTx2783 (resistant)] plants and chi-square analysis for a single, dominant gene for resistance.

	Observed		Expected (3:1)			
Number of F_2_ plants	R	S	R	S	χ^2^	*p*-value
160	128	32	120	40	2.13	0.144


*R, resistant parent phenotype and S, susceptible parent phenotype.*

### Sugarcane Aphid Preference

**FIGURE 10:**
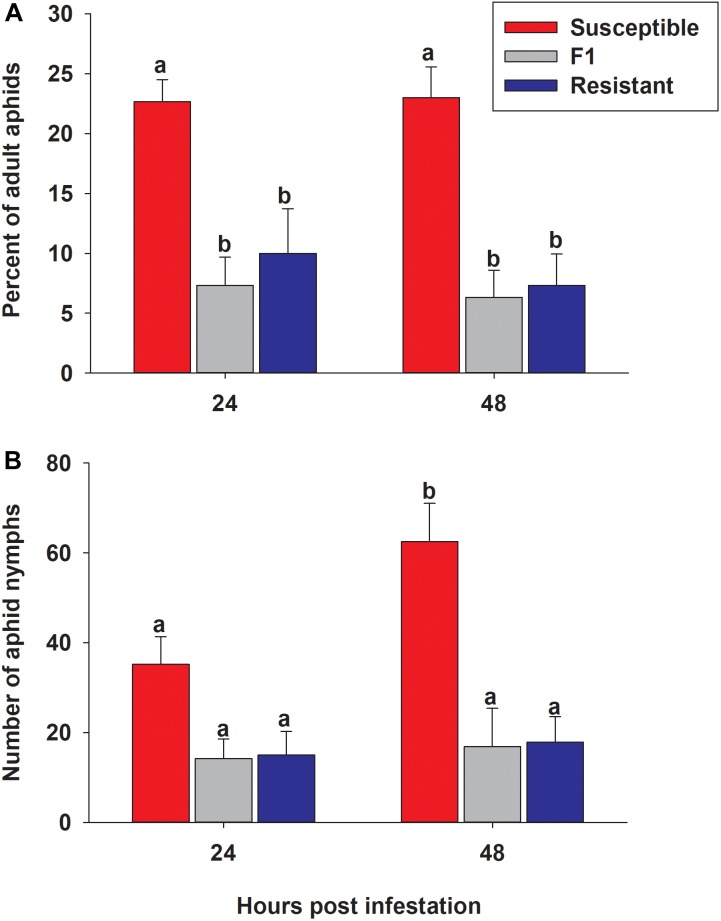
Sugarcane aphid preference on susceptible (BCK60), resistant (RTx2783) and F_1_ (ACK60 × RTx2783) sorghum plants. **(A)** The percentage of adult aphids on each genotype after 24 and 48 h post infestation (genotype: *p* < 0.0001; time: *p* = 0.6081 genotype × time: *p* = 0.8497), **(B)** the number of nymphs produced during preference experiment (genotype: *p* < 0.0001; time: *p* = 0.0338 genotype × time: *p* = 0.0784). Values presented are least square means (±1 SE). Bars with the same letter are not significantly different from one another at α ≤ 0.05 using Tukey’s HSD test.

To determine whether aphids showed a feeding preference for either the resistant or susceptible line, 50 adult aphids were presented with a choice between resistant, susceptible and F_1_ plants. Numbers of adult aphids present and nymphs produced on each line at 24 and 48 h post infestation were significantly different among the three lines (*p* < 0.0001 for both timepoints) (Figure 10). The percent of the original 50 adult aphids observed on the resistant and F_1_ plants was significantly lower than susceptible plants at both 24 h (10.0% ± 3.7, 7.3% ± 2.4, and 22.7% ± 1.8, respectively) and 48 h (7.3% ± 2.6, 6.3% ± 2.3, and 23.0% ± 2.6, respectively) (Figure 10A). There were no significant differences between time (24 and 48 h) or genotype × time interaction in the percent of adult aphids on sorghum lines (*p* = 0.6081 and *p* = 0.8497, respectively; Figure 10A). There was a significantly higher number of nymphs observed over the time course (*p* = 0.0338) on the susceptible line than both the resistant and F_1_ hybrid plants with 35.2 ± 6.1, 15.0 ± 5.2, and 14.2 ± 4.4, at 24 h, and at 48 h 62.5 ± 8.5, 17.8 ± 5.7, and 16.8 ± 8.5, respectively (Figure 10B). These results suggested that sugarcane aphid has a preference for the susceptible plants over the resistant and F_1_ plants and there are adverse effects on aphid biology with a decrease in nymph production on the resistant and F_1_ plants.

### Evaluation of Sugarcane Aphid Feeding Behavior on Resistant and Susceptible Plants

**FIGURE 11:**
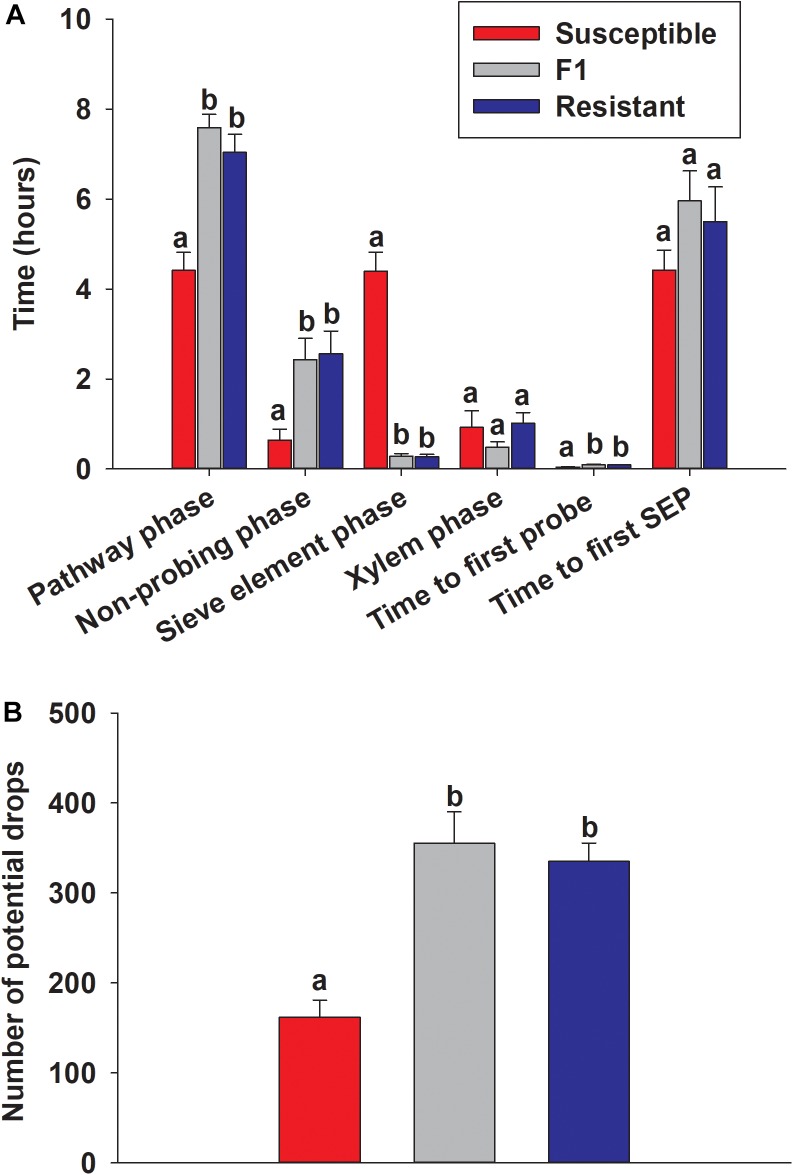
EPG recordings for the sugarcane aphid on the susceptible (BCK60), F_1_ (ACK60 × RTx2783) and resistant (RTx2783) sorghum plants. **(A)** Time spent by the sugarcane aphid for different phases on leaf surface during 10 h of EPG monitoring. **(B)** Number of potential drops (brief intracellular punctures) during 10 h for the sugarcane aphid on each sorghum line. Values presented are least square means (±1 SE). Bars with the same letter are not significantly different from one another within EPG category at α ≤ 0.05 using Tukey’s HSD test. SEP, sieve element phase.

EPG used to assess the probing behavior of adult sugarcane aphid on resistant, susceptible and F_1_ plants. The proportions of time that tethered aphids spent penetrating between cells to vascular tissue (pathway phase), non-probing phase, time to first probe and time spent in phloem sieve element phase was significantly different between the resistant and the susceptible plants, but not significantly different between resistant and F_1_ plants (Figure 11). The aphids spent significantly longer time in the pathway phase of the resistant and F_1_ plants (7.04 ± 0.40 and 7.59 ± 0.30 h, respectively) compared to the susceptible line (4.41 ± 0.40 h) (Figure 11A), which suggested the presence of resistance factors hindering the ability of aphids to successfully locate the vascular tissues (*p* < 0.0001). Furthermore, once aphids reach the phloem tissues, aphids were not able to feed continuously in the phloem for extended periods on the resistant and F_1_ plants (0.27 ± 0.05 and 0.29 ± 0.05 h, respectively), compared to the susceptible line (4.40 ± 0.42 h; *p* < 0.0001) (Figure 11A). Similarly, aphids spent significantly less time in non-probing phase while feeding on the susceptible line BCK60 (0.65 ± 0.24 h) compared to the resistant and F_1_ plants (2.56 ± 0.50 and 2.43 ± 0.46 h, respectively; *p* < 0.0003) (Figure 11A). Significant differences in the time the aphids spent in the xylem phase were not observed and were comparable among all three lines (*p* < 0.1131). Potential drops, which are the brief intracellular punctures during the pathway phase, were significantly higher with the resistant and F_1_ plants (334.87 ± 20.46 and 354.87 ± 35.21, respectively) than the susceptible line (161.75 ± 18.92; *p* < 0.0001) (Figure 11B). Taken together, our results suggest that the surface features or cell wall properties in addition to the factors present in the phloem sap may play a role in resistance mechanism of RTx2783. Further, there was a significantly longer duration in the “time to first probe” observed on resistant plants than the susceptible ones (*p* < 0.0001) (Figure 11A).

## Discussion

The responses to sugarcane aphid herbivory between the aphid-resistant RTx2783 and the aphid-susceptible BCK60 sorghum parental lines were characterized through a series of experiments to determine how RTx2783 affected insect behavior and fecundity compared to the susceptible line and how plant development and plant transcriptional responses to aphid feeding differed between the two lines.

The inheritance of sugarcane aphid resistance in a cross between the susceptible and resistant lines used in the study fits a model of a single dominant locus (Table 2). In other systems, single copy dominant genes often confer aphid resistance; for example, the *Meu-1* gene in tomato confers resistance to the potato aphid (*Macrosiphum euphorbiae*) (Rossi et al., 1998), the *Vat* gene confers resistance to cotton-melon aphid (*Aphis gossypii*) in muskmelon (*Cucumis melo*) (Dogimont et al., 2010), and *Rag1* confers resistance to the soybean aphid (*Aphis glycines*) in soybean (*Glycine max*) (Hill et al., 2006). Other examples of dominant, single gene aphid resistance include resistance in apple (*Malus* spp.) to the rosy leaf-curling aphid (*Dyaphis devecta*), resistance in peach (*Prunus persicae*) to the green peach aphid (*Myzus persicae*), resistance in *Medicago truncatula* to the blue alfalfa aphid (*Acyrthosiphon kondoi*) and resistance in cowpea (*Vigna unguiculata*) to the cowpea aphid (*Aphis craccivora*) (Alston and Briggs, 1977; Bata et al., 1987; Pascal et al., 2002; Klingler et al., 2005). *Meu-1* was one of the first isolate-specific insect resistant genes to be cloned and belongs to the NBS-LRR family of resistance genes (Rossi et al., 1998). Similarly, the *Vat* gene was isolated by a map-based cloning strategy and was found to encode an NBS-LRR (Pauquet et al., 2004), and several other aphid resistant genes have been mapped to an NBS-LRR gene cluster (Klingler et al., 2005; Hill et al., 2006; Kim et al., 2010). Most of the resistant genes that have been identified in plants encode NBS-LRRs. The transcriptome comparisons between resistant and susceptible control plants highlighted several NBS-LRR and disease resistance proteins that were increased in the resistant line and there are several NBS-LRR and disease resistance proteins identified in the resistant and susceptible plants early (day 5) in the network analysis, which suggests an NBS-LRR may confer sugar aphid resistance in RTx2783. There are also instances of recessive genes that confer resistance to aphids in crop species. For example the *dn3* gene in *Triticum tauschii* is recessive and confers resistance to the Russian wheat aphid (*Diuraphis noxia*) (Nkongolo et al., 1991). Other examples for recessive, single gene aphid resistance include resistance in maize to the corn aphid (*Rhopalosiphum maidis*) and in peanut (*Arachis hypogea*) to the cowpea aphid *(Aphis craccivora*) (Carena and Glogoza, 2004; Herselman et al., 2004). Although the molecular mechanisms and identity of genes that confer recessive resistance to aphids are not as well characterized as those associated with genes inherited in a dominant manner, one idea proposed is that these resistance-associated alleles encode for proteins that trigger the interruption of efficient aphid feeding (Dogimont et al., 2010). In the case of RTx2783, introgression of the resistance gene into sorghum germplasm will be relatively straightforward because of its simple mode of inheritance and easily distinguishable resistance in bioassays.

The results of sugarcane aphid feeding assays indicate that the resistant line RTx2783 displays both antixenosis and antibiosis modes of resistance. In choice assays, sugarcane aphids exhibited a strong preference for the susceptible line instead of either the resistant or F_1_ hybrid plant (Figure 10A). After 48 h, fewer nymphs were counted on the resistant and F_1_ plants than the susceptible host (Figure 10B). Host selection by adult aphids is normally the first stage of colonization and plays a major role in determining aphid populations in the field. Sugarcane aphids avoid RTx2783, which is likely an important part of its aphid resistance. The EPG analysis indicated that the insects spent less time feeding from the sieve elements of the resistant or F_1_ plants than the susceptible plants (Figure 11A), which suggests the phloem contents could be involved in deterring feeding on resistant plants. The fewer nymphs on the resistant and F_1_ plants may have resulted from either antibiosis where host factors reduce the fecundity of pest, or antixenosis where host factors alter the feeding behavior of pest that in return affects fecundity.

Existence of resistance factors in phloem have been previously suggested in different types of plant–aphid interactions (Chen et al., 1997; Klingler et al., 1998; Klingler et al., 2005; Diaz-Montano et al., 2007). A main component of induced aphid-resistance is often derived from phloem factors (Diaz-Montano et al., 2007; Crompton and Ode, 2010). Phloem resistant factors include: phloem protein coagulation and callose deposition (Tjallingii, 2006), accumulation of lectins (Down et al., 1996; Gatehouse et al., 1997), protease inhibitors (Tran et al., 1997; Kehr, 2006), and secondary metabolites (Dinant et al., 2010). Network analysis of the transcriptomes showed KEGG gene sets related to carbohydrate metabolism decreased first at day 5 in the resistant line and later at day 10 in the susceptible line, which could be acting maintain phloem sucrose concentration in response to aphid feeding. These phloem resistance factors can contribute to antixenosis by restricting duration of aphid feeding (Will et al., 2013) or antibiosis by adversely affecting aphid fecundity (Ribeiro et al., 2006; Sylwia et al., 2006; Goławska, 2007). Network analysis revealed a phloem protein 2 (PP2) gene that was increased with aphid infestation, in pumpkin (*Cucurbita maxima)* PP2 along with phloem protein 1 (PP1) are synthesized in companion cells and transported into sieve elements via plasmodesmata (Bostwick et al., 1992). In pumpkin, PP2 is also suggested to function as a pathogen (bacteria and fungi) removal protein in damaged phloem sieve elements (Read and Northcote, 1983) and under certain conditions PP2 can enter the phloem long-distance transport stream where it is proposed to seal sieve element pores upon wounding (Alosi et al., 1988; Walz et al., 2004). Increased expression of this gene could indicate a defense within sieve elements against aphid feeding. Our results for the sugarcane aphid in the choice assay and EPG feeding behavior assay on the susceptible, resistant and F_1_ plants suggest antixenosis is the predominant mode of resistance in RTx2783; however, antibiosis is also a possibility. The resistance gene in RTx2783 may be similar to the potato aphid resistant gene *Mi*, which belongs to the NBS-LRR resistance genes, and also confers both antixenosis and antibiosis (Rossi et al., 1998). Future experiments investigating phloem contents and sugarcane aphid life history traits are needed to further tease apart antibiosis and antixenosis mechanisms.

The EPG technique also may identify host tissues that could play a role in host resistance mechanisms. Surface resistance is the first line of defense against attack, and a long time to first probe reflects the effects of mechanical barriers or olfactory repellents present at the leaf surface, such as trichomes or toughness of the leaf surface (Van Helden and Tjallingii, 1993). Potential surface resistance was observed in the resistant and F_1_ sorghum plants, because time to first probe was significantly longer in the resistant and F_1_ plants in comparison to the susceptible plants. Longer durations in “time to first probe” have previously been associated with aphid (*Myzus persicae*)-feeding difficulties at the leaf surface of potato (*Solanum*) species (Alvarez et al., 2006). Additionally, almost twice as much time passed before the first phloem event (pathway phase) and time spent in the non-probing phase for aphids reared on the resistant and F_1_ plants compared to the susceptible plants. These observations may indicate that the sugarcane aphid encounters resistance factors on their way to the phloem in both the resistant and F_1_ plants, which has also been observed previously in a lettuce line that displays resistance to the black currant-lettuce aphid (Ten Broeke et al., 2013). Thus, these data suggest that the factors involved in aphid resistance are found not only in the phloem, but also at the surface of the leaf and pathway to phloem. The transcriptional response of the RTx2783 resistant plants showed only a few genes differentially expressed as a result of aphid infestation, which indicated the plants both perceived substantially less stress and sustained less damage resulting from sugarcane aphid feeding.

However, network analysis uncovered broad patterns of co-expressed genes in the resistant plants that were related to hormone signal transduction, phenylpropanoid biosynthesis, carbon metabolism, carbohydrate metabolism and plant pathogen KEGG pathways, although in a few instances similar patterns were also observed with the susceptible line. In contrast, DEGs identified in the BCK60 susceptible plants upon infestation were typical of herbivory or biotic stresses, which included genes involved in photosynthesis, sugar transport, phytohormones and wound responses (Kessler and Baldwin, 2002; Studham and Macintosh, 2013; Prochaska et al., 2015). As early as day 5 post aphid infestation significant differences in the transcriptomes of sugarcane aphid infested and uninfested plants were apparent in the susceptible line. Changes in the expression levels of genes related to photosynthesis, starch catabolism, sucrose metabolism and sugar transport were among the early signs of defense responses. Notably, suppression of photosynthesis is a universal early response to aphid feeding (Kerchev et al., 2012; Donze-Reiner et al., 2017). However, plants that are able to maintain photosynthesis under insect attack often exhibit greater resistance (Haile et al., 1999; Zhu-Salzman et al., 2004; Franzen et al., 2007; Gutsche et al., 2009). In this regard the resistant line, RTx2783 responded with small reductions in expression levels of photosynthesis related genes early at day 5, but photosynthesis-related gene expression recovered by day 10, which appears to be similar to other aphid resistant plants.

The plant hormones JA and ethylene play key roles as signaling molecules in abiotic and biotic stress responses, which include plant-aphid interactions. In susceptible soybean plants, aphids suppressed the jasmonate signaling of the plant (Studham and Macintosh, 2013). In contrast, sugarcane aphid herbivory elicited a large-scale defensive response in the susceptible BCK60 sorghum plants that included the upregulation of genes involved in JA and ethylene biosynthesis (Figure 4). In Arabidopsis, ethylene response factor 6 (*ERF6)* plays an important role as a positive antioxidant regulator in response to biotic stresses (Sewelam et al., 2013). Interestingly, two ethylene responsive element binding transcription factor were up-regulated and increased in the network analysis in the resistant RTx2783 sorghum line at day 5 post sugarcane aphid infestation, which may indicate this gene product could be involved in its resistance.

Reactive oxygen species (ROS) are a well-recognized component of plant response to insect herbivory (Kerchev et al., 2012; Foyer and Noctor, 2013), and several studies suggest tolerant plants have a greater ability to quench excess ROS (Heng-Moss et al., 2004; Franzen et al., 2007; Ramm et al., 2015). Enzymes such as peroxidases and glutathione-S-transferases (GSTs) reduce ROS accumulation and detoxify oxidized metabolites and xenobiotic when plants encounter reactive oxygen generating stressors (Gulsen et al., 2010;Perez and Brown, 2014). In the current study, the susceptible line, BCK60 has a substantial number of peroxidase genes whose expression was down-regulated in the infested plants. This result aligns with previous observations that insect susceptible plants generally have a lower capacity to detoxify ROS and its downstream products in comparison to plants with resistance (Smith et al., 2010; Sytykiewicz et al., 2014). In sorghum, increased expression of a peroxidase gene cluster has been associated with resistance to the aphid greenbug biotype I (Scully et al., 2016). Induction of detoxification genes has emerged as a trend in tolerant plants to potentially counteract deleterious effects of hemipteran herbivory (Koch et al., 2016). In the resistant line RTx2783, an up-regulation of detoxifying genes was not observed, which suggests its resistance mechanisms align with antixenosis and antibiosis and not tolerance. The global transcriptional response of the susceptible line BCK60 to sugarcane aphid infestation was consistent with the damage observed in the caged plant experiment and indicative of plant stress responses to insect herbivory; while the resistant RTx2783 line exhibited little gene expression differences due to aphid infestation, which supports the ability of this line to maintain aphid populations and sustain little damage.

In summary, our results indicate a single dominant locus confers resistance in RTx2783, which was linked to increased expression of classical resistance gene-like sequences (NBS-LRRs). The underlying resistance mechanisms in RTx2783 to sugarcane aphids appear to include both antixenosis and antibiosis. Gene expression analysis demonstrated the resistant line does not display the same expression levels of stress-related genes during sugarcane aphid infestation as the susceptible line, instead the resistant line appears to progress normally through plant development based upon global gene expression. The expression of several wound response and phloem defense genes were also induced upon aphid infestation in the RTx2783, which may confer antixenosis. Preference and EPG studies showed that sugarcane aphid avoid and reduced its feeding on the resistant line, respectively, which are indicative of antixenosis. The decreased production of sugarcane aphid nymphs on the resistant line provides evidence for antibiosis; however, further studies are needed to determine whether there are also negative impacts to aphid physiology, mortality, reproduction, fitness or rates of growth or development. RTx2783 represents one source of resistance that could be introgressed into a wide range of R-lines for the production of grain and forage hybrids with durable resistance to sugarcane aphid.

## Author Contributions

HT and SS designed the research. HT, SG, ES, and TG performed the experiments. HT, SG, ES, TG, NP, GS, JL, and SS analyzed and interpreted the data. HT and SS wrote the first draft of the manuscript. All authors reviewed and revised the manuscript prior to publication.

## Conflict of Interest Statement

The authors declare that the research was conducted in the absence of any commercial or financial relationships that could be construed as a potential conflict of interest.

## Footnotes

1 http://www.epgsystems.eu/

2 https://www.unmc.edu/vcr/cores/vcr-cores/genomics/next-generation/index.html

3 phytozome.jgi.doe.gov/pz/portal.html

4 www.genome.jp/kegg/pathway.html
