# Supplementary material for: Global Responses of Resistant and Susceptible Sorghum (Sorghum bicolor) to Sugarcane Aphid (Melanaphis sacchari)
Source: Front Plant Sci. 2019 Feb 22;10:145. doi: 10.3389/fpls.2019.00145 (PMC6396740; doi:10.3389/fpls.2019.00145)
Supplement: Supplementary file 5 [file Data_Sheet_1.PDF]

Supplementary Material

Global responses of resistant and susceptible sorghum (*Sorghum bicolor*) to sugarcane aphid (*Melanaphis sacchari*)

Hannah M. Tetreault[1,2], Sajjan Grover[3], Erin D. Scully[1,2‡], Tammy Gries[1], Nathan Palmer[1,2], Gautam Sarath[1,2], Joe Louis[3] and Scott E. Sattler[1,2*]

[1]Wheat, Sorghum and Forage Research Unit, USDA-ARS, Lincoln, NE 68583, USA

[2]Department of Agronomy and Horticulture, University of Nebraska-Lincoln, Lincoln, NE 68583, USA

[3]Department of Entomology, University of Nebraska-Lincoln, Lincoln, NE 68583, USA

*Correspondence:

Dr. Scott Sattler

scott.sattler@ars.usda.gov

Supplementary Figures

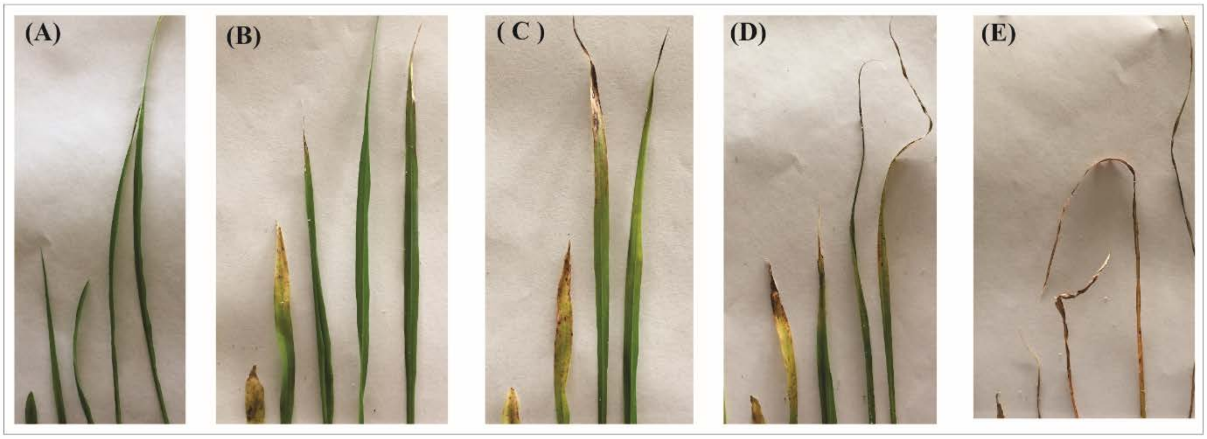

Supplementary Figure 1. Plant damage on a scale of 1 to 5. Plant damage was rated based on amount of leaf discoloration and leaf rolling. The amount of damage was quantified using a 1 to 5 scale adapted from Heng-Moss et al. (2002). Damage score of **(A)** 1,-plants appear healthy, may have small spots of discoloration, **(B)** 2, discoloration and leaf rolling comprising 20-39% of total leaf area, **(C)** 3, discoloration and leaf rolling that comprised 40-59% of total leaf area, **(D)** 4, discoloration and leaf rolling that is 60-79% of total leaf area and **(E)** 5, plants appear dead, discoloration and leaf rolling that is 80-100% of total leaf area.

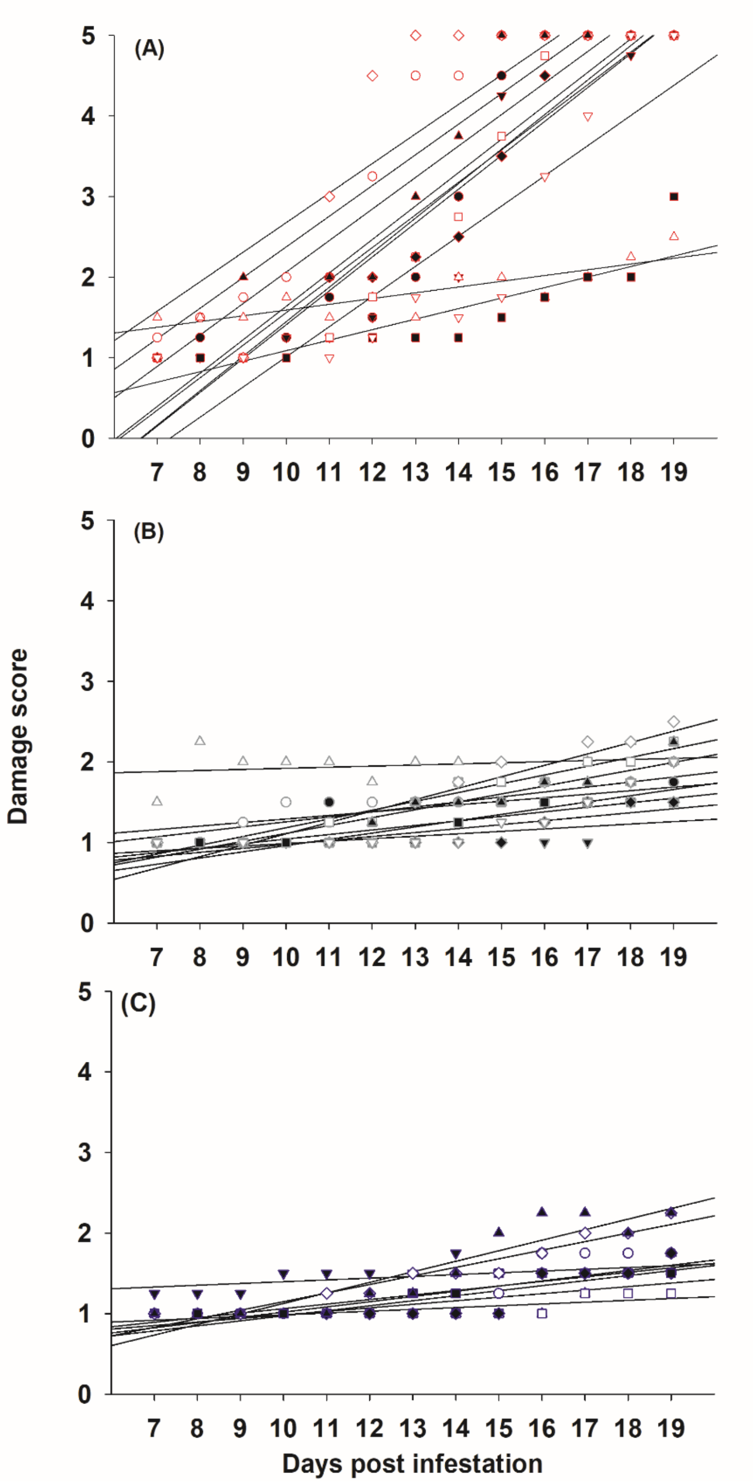

Supplementary Figure 2. Damage scores for (A) susceptible (A/BCK60), (B) F_1 (A/BCK60 x RTx2783) and (C) resistant (RTx2783) sorghum plants 7 days post infestation to 19 days post infestation in an open tray evaluation of sugarcane aphid damage. Linear regression implemented in JMP 12.2.0 (SAS Institute Inc.) to determine the line of best fit for each plant over the time course and slope was used for the rate of change in damage for binning individuals in Figure 11A. Shapes indicate a single individual within genotype.

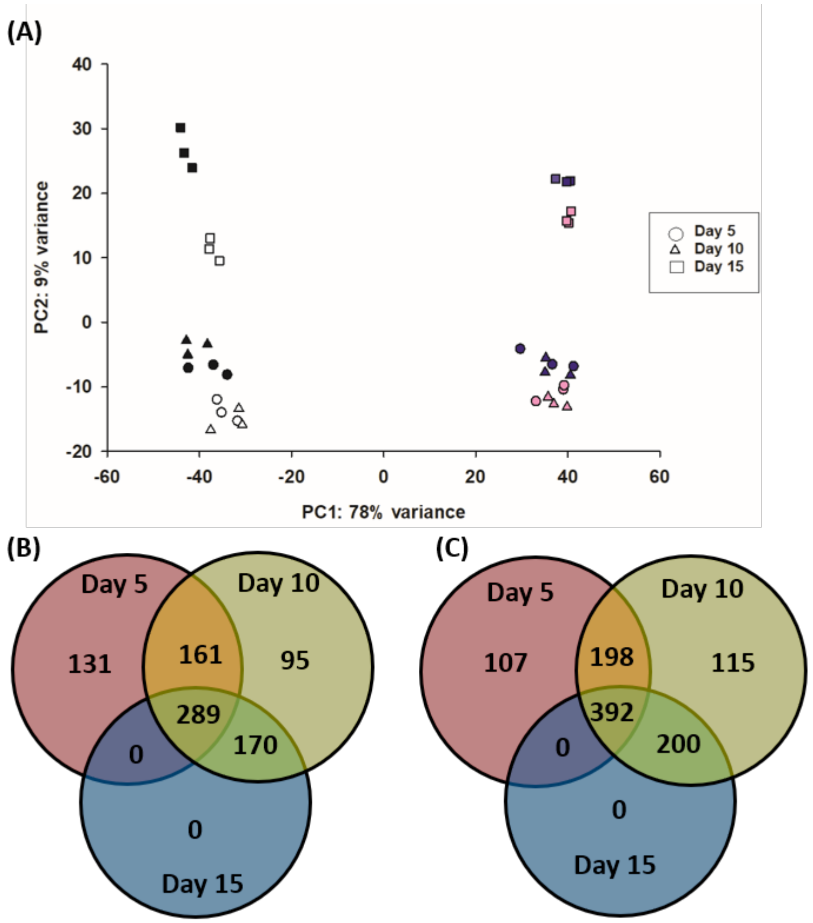

Supplementary Figure 3. Overview of differentially expressed across days for the resistant (RTx2783) control plants relative to the susceptible (A/BCK60) control plants. (A) Principal component analysis of RNA-seq data on individual samples from all RNA-seq samples. Venn diagrams of genes **(B)** increased in susceptible relative to resistant control plants and **(C)** decreased in susceptible relative to resistant control plants. PCA marker colors, black = susceptible infested, white = susceptible control, blue= resistant infested and pink = resistant control.
